# Autochthonous Leishmaniasis Caused by *Leishmania tropica*, Identified by Using Whole-Genome Sequencing, Sri Lanka

**DOI:** 10.3201/eid3009.231238

**Published:** 2024-09

**Authors:** Hermali Silva, Tiago R. Ferreira, Kajan Muneeswaran, Sumudu R. Samarasinghe, Eliza V.C. Alves-Ferreira, Michael E. Grigg, Naduviladath V. Chandrasekharan, David L. Sacks, Nadira D. Karunaweera

**Affiliations:** University of Colombo, Colombo, Sri Lanka (H. Silva, K. Muneeswaran, S.R. Samarasinghe, N.V. Chandrasekharan, N.D. Karunaweera);; Laboratory of Parasitic Diseases, National Institutes of Health, Bethesda, Maryland, USA (T.R. Ferreira, E.V.C. Alves-Ferreira, M.E. Grigg, D.L. Sacks)

**Keywords:** *Leishmania tropica*, *Leishmania donovani*, leishmaniasis, autochthonous, cutaneous, mucocutaneous, parasites, zoonoses, vector-borne infections, whole-genome sequencing, mucosal, Sri Lanka

## Abstract

Cutaneous leishmaniasis is atypical in Sri Lanka because *Leishmania donovani*, which typically causes visceral disease, is the causative agent. The origins of recently described hybrids between *L. donovani* and other *Leishmania* spp. usually responsible for cutaneous leishmaniasis remain unknown. Other endemic dermotropic *Leishmania* spp. have not been reported in Sri Lanka. Genome analysis of 27 clinical isolates from Sri Lanka and 32 Old World *Leishmania* spp. strains found 8 patient isolates clustered with *L. tropica* and 19 with *L. donovani*. The *L. tropica* isolates from Sri Lanka shared markers with strain *Lt*K26 reported decades ago in India, indicating they were not products of recent interspecies hybridization. Because *L. tropica* was isolated from patients with leishmaniasis in Sri Lanka, our findings indicate *L. donovani* is not the only cause of cutaneous leishmaniasis in Sri Lanka and potentially explains a haplotype that led to interspecies dermotropic *L. donovani* hybrids.

Leishmaniasis is a heterogeneous group of diseases caused by protozoan parasites of the genus *Leishmania*, transmitted by the bite of phlebotomine sand flies (*1*). The 3 typical clinical presentations of leishmaniasis affecting humans are visceral leishmaniasis (VL), cutaneous leishmaniasis (CL), and mucocutaneous leishmaniasis (MCL) (*1*). Those who are most affected are persons in Asia, Africa, and Latin America who suffer from poverty (*2*).

Although *Leishmania donovani* causes VL in other countries in Asia and Africa, an atypical variant of the same species is almost exclusively associated with CL in Sri Lanka (*3*). Common symptoms of CL are papules, nodules, plaques, and ulcers. Leishmaniasis cases in Sri Lanka have increased over the past 2 decades, from 22 in 2001 to 3,389 in 2022 (https://www.epid.gov.lk). Over the past 2 decades, <10 cases of leishmaniasis have been VL in Sri Lanka; some cases have included severe chronic conditions preceding or occurring after the *Leishmania* infection (*4*). There are 2 primary hotspots in Sri Lanka, 1 in the southern province and 1 in the north-central province (*5*).

The genetic factors associated with disease phenotypes of leishmaniasis are not well understood. The A2 multigene family in *Leishmania* might be associated with either dermotropic or viscerotropic phenotypes (*6*). A2 gene variants encode stress-induced transmembrane proteins that are key for the parasite tropism to internal organs observed in visceralizing species (*6*). Abnormal changes in the number of chromosome copies that characterize extensive aneuploidy, usually harmful in most complex organisms, are frequent and highly tolerated in *Leishmania* parasites because they have a role in gene expression modulation (*7*,*8*).

The leishmaniasis disease profile worldwide is typically associated with the causative species of *Leishmania* (*9*). However, descriptions of emerging isolates and atypical phenotypes have made this association less clear (*6*,*10*). Recent genetic studies have shown that the *Leishmania* natural population structure is more complex than previously thought, partially because of the plasticity of the parasite genome and the occurrence of sexual reproduction (*11*). Isolates from the same clade might carry relevant genetic features accounting for shifts in clinical phenotypes. For example, *L. donovani* isotype MON-37 (from the Montpellier typing system) is the known causative agent of CL in Sri Lanka, and MON-2 is the known causative agent of VL in India (*3*,*12*).

A more recent analysis of *L. donovani* clinical isolates from Sri Lanka reported interspecies genomic hybrids between *L. donovani* and 2 common cutaneous species, *L. major* from Africa and *L. tropica* from the Middle East (*13*). The evidence of hybridization and introgression history in the *Leishmania* population in Sri Lanka suggests genetic exchange might have played a role in the insurgence of dermotropic *L. donovani*. However, many epidemiologic aspects of this model are unclear. Most CL causing *L. donovani* isolates described to date do not display clear evidence of hybridization with dermotropic species, and the parental strains of possible hybrid parasites in Sri Lanka remain unknown. Currently, <20 high quality next-generation sequencing (NGS) datasets of *Leishmania* spp. isolated from patients in Sri Lanka are publicly available (*13*). Therefore, it is crucial to perform whole-genome sequencing (WGS) analysis on a wider range of clinical isolates to better understand the atypical pathogenesis of leishmaniasis in Sri Lanka.

We studied WGS results of 27 *Leishmania* clinical isolates from Sri Lanka and made multiple genetic comparisons by using 32 different Old World *Leishmania* strains, including 5 interspecies *L. donovani* hybrids previously reported in Sri Lanka. Among the genomes analyzed, we describe autochthonous *L. tropica* isolates in patients from Sri Lanka who, apart from 1 exception, did not have travel history outside the country.

This study has been approved by the Ethics Review Committee, Faculty of Medicine, University of Colombo, Sri Lanka (approval no. EC-16-080). Written, informed consent was obtained from the participants.

## Methods

### *Leishmania* Culture

We cultured *Leishmania* promastigotes from 27 lesion aspirates (26 CL and 1 MCL) in M199 culture medium supplemented with 10% heat-inactivated fetal bovine serum (Thermo Fisher Scientific, https://www.thermofisher.com) and 100 IU/ml of penicillin and 100mg/ml of streptomycin (Thermo Fisher Scientific). We incubated the cultures at 25 ± 1°C until the logarithmic phase promastigote count reached 10^7^/mL and then harvested them. 

### Genomic DNA Isolation and Sequencing

We extracted DNA from the cultured *Leishmania* promastigotes by using a QIAamp DNA Mini Kit (QIAGEN, https://www.qiagen.com) and prepared libraries by using the Nextera XT DNA library preparation kit (Illumina, https://www.illumina.com) and the Nextera DNA Flex library preparation kit (Illumina), according to the manufacturer’s instructions. Paired-end sequencing was conducted by Applied Biological Materials (British Columbia, Canada) by using the NextSeq (2×75bp) and HiSeq 4000 (2×150bp) platforms (Illumina).

### WGS Data Analysis

**Figure 1:**
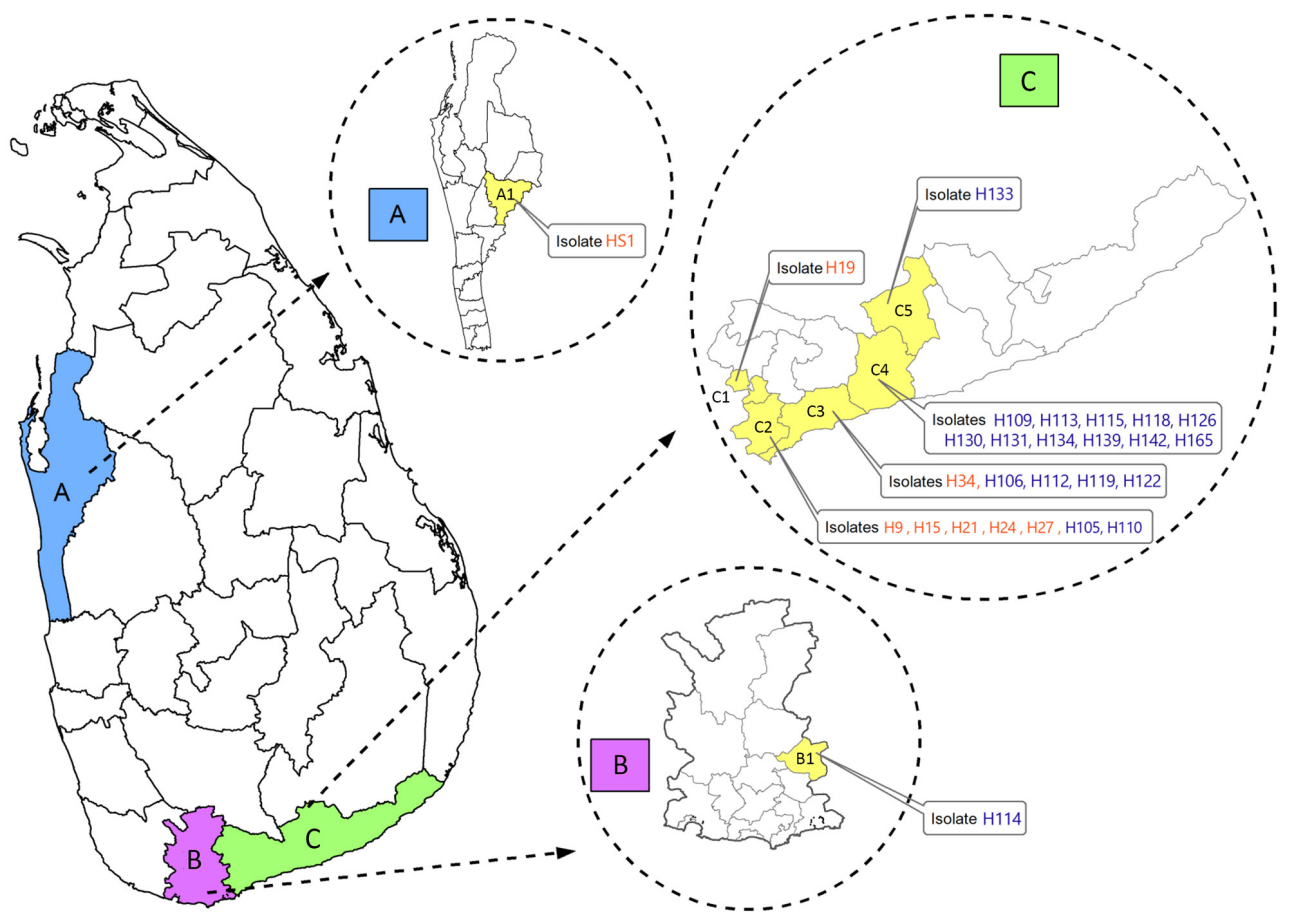
Geographic locations of patients with cutaneous or mucocutaneous leishmaniasis in Sri Lanka. The mucocutaneous leishmaniasis patient was from the Puttalam district (A), Anamaduwa subdistrict. The cutaneous leishmaniasis patients were from both the Matara (B) and Hambantota (C) districts. Isolates labeled in orange were identified as *Leishmania tropica*. Isolates labeled in blue were identified as *L. donovani*. A, Puttalam district; A1, Anamaduwa; B, Matara district; B1, Hakmana; C, Hambantota district; C1, Okewela; C2, Beliatta; C3, Tangalle; C4, Ambalantota; C5, Sooriyawewa.

We analyzed WGS data of 27 clinical isolates from this study and 32 *Leishmania* genomes of Old World species available from the National Center for Biotcechnology Information Sequence Read Archive (https://www.ncbi.nlm.nih.gov/sra) or the European Nucleotide Archive (https://ebi.ac.uk/ena). We detected genome-wide single-nucleotide polymorphisms (SNPs) in each sample after mapping high-quality reads to the reference strain, *L. tropica* L590 or *L. donovani* CL–Sri Lanka (genome available on TriTrypDB, https://tritrypdb.org). We extracted the consensus sequences of 7 different genetic markers to identify the clinical isolates at the species level. Phylogenetic analysis by NGS multilocus sequence typing (MLST) involved 59 genomes of nucleotide sequences with the indicated number of base pairs from the following genes: ribosomal RNA internal transcribed spacer (ITS; *L. donovani* CL–Sri Lanka CP029526:1015798–1016063), 265 bp; glucose-6-phosphate dehydrogenase (*G6PD*; LtrL590_34:26953–27953), 1,001 bp; glucose-6-phosphate isomerase (*GPI*; LtrL590_12:302579–303479), 901 bp; isocitrate dehydrogenase precursor (*ICD*; LTRL590_SCAF000112:124407–125407), 1,001 bp; phosphomannose isomerase (*PMI*; LtrL590_32:632360–633360), 1,001 bp; aspartate aminotransferase (*AST*; LtrL590_35:290874–291838), 965 bp; and inosine-guanine nucleoside hydrolase (*IGNH*; LtrL590_14:44174–44933), 760 bp (*14*). We extracted the ITS reference sequence from the *L. donovani* CL–Sri Lanka reference genome because the ITS sequence is not fully assembled in *L. tropica* L590. We conducted phylogenetic analysis with maximum-likelihood method and tamura-nei model by using NGS MLST sequences as input in MEGA X (*15*). We compiled the pileup of read alignments to each locus used for 3 representative *L. tropica* genomes (Appendix 1 Figure 1). We checked the aneuploidy profiles by inferring the chromosome copy values from whole-genome analysis normalized read depth after we mapped to the *L. tropica* L590 reference genome v.57, found in TriTrypDB, and assuming that all samples are diploid.

To confirm the absence of species admixture in the new *L. tropica* from Sri Lanka, we studied the genomewide zygosity profiles and the population genetic structure by tracking SNP frequencies in all 59 WGS samples. After mapping, we used aligned reads to identify SNPs in heterozygosity (allele frequency 0.15–0.85) or in homozygosity (allele frequency >0.85) by using the PAINT software suite (*16*). We investigated the gene sequences of the A2 virulence factor (*L. donovani* CL–Sri Lanka CP029521:270000–320000) to identify any association with the phenotype of CL. We generated pseudo-sequences from 1,368,585 high-quality whole-genome linked SNPs that had information in >50% of the samples to build phylogenetic network trees in SplitsTree v.6.0.2.3 (https://software-ab.cs.uni-tuebingen.de/download/splitstree6/welcome.html) (*17*). We calculated hamming distances and used the neighbor-net method to generate a splits network. A total of 1,000 bootstrap split replicates were used for internode confirmation of the phylogenetic tree of the *L. tropica* genomes, with a minimum 50% cutoff.

### Data Availability

Data supporting the conclusions of this article are within the article and Appendix files, including Sequence Read Archive accession numbers for all the genomic data used in this work (Appendix 2 Tables 1–3). We deposited raw sequencing data in the National Center for Biotechnology Information BioProject archive (accession no. PRJNA904745).

## Results

We recovered *Leishmania* clinical isolates from 27 patients in 3 administrative districts in Sri Lanka: Hambantota (n = 25), Matara (n = 1), and Puttalam (n = 1) (Figure 1). The CL patients were from the southern region and the MCL patient was from the north-western region of the country. Clinical manifestations of the CL lesions consisted of 8 papules, 3 plaques, and 15 ulcers. Eight of the ulcers were ulcerated nodules (Appendix 2 Table 4).

### Species Variability among Clinical Isolates

**Figure 2:**
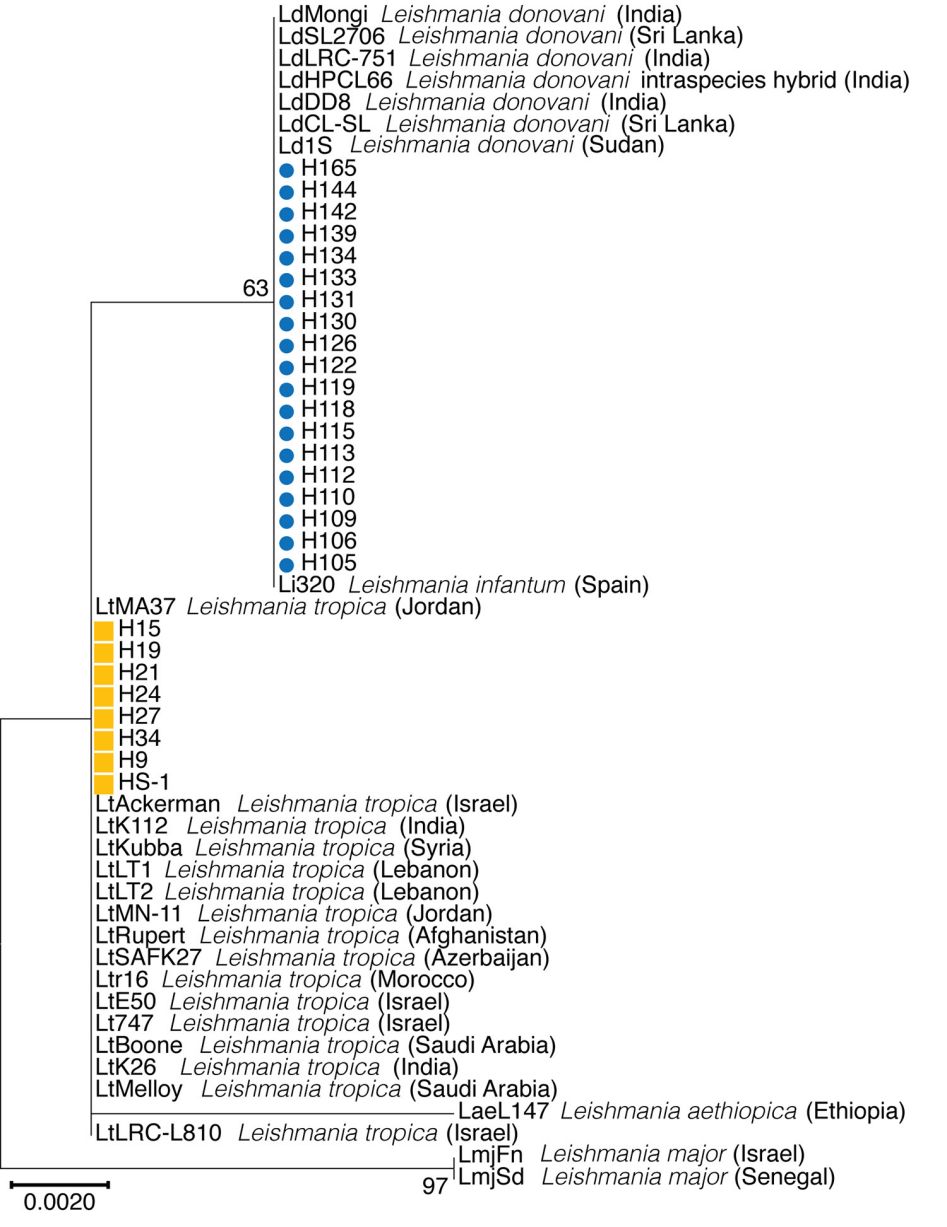
Phylogenetic analysis of *Leishmania* spp. clinical patient isolates from Sri Lanka and reference *Leishmania* spp. strains using sequences of the ribosomal RNA internal transcribed spacer (ITS). Sri Lanka isolates (H and HS) form 2 groups, 1 co-cluster with *L. donovani* (blue circles) and 1 with *L. tropica* (orange squares). Maximum-likelihood method and Tamura-nei models were performed for phylogenetic analysis using MEGA X (*15*). For each gene, the phylogenetic tree with the highest log likelihood (−429.25p for ITS) is presented from 1,000 bootstrap replicates. Bootstrap percentages >55% are shown for each branch. Scale bar represents the number of mutations per site.

**Figure 3:**
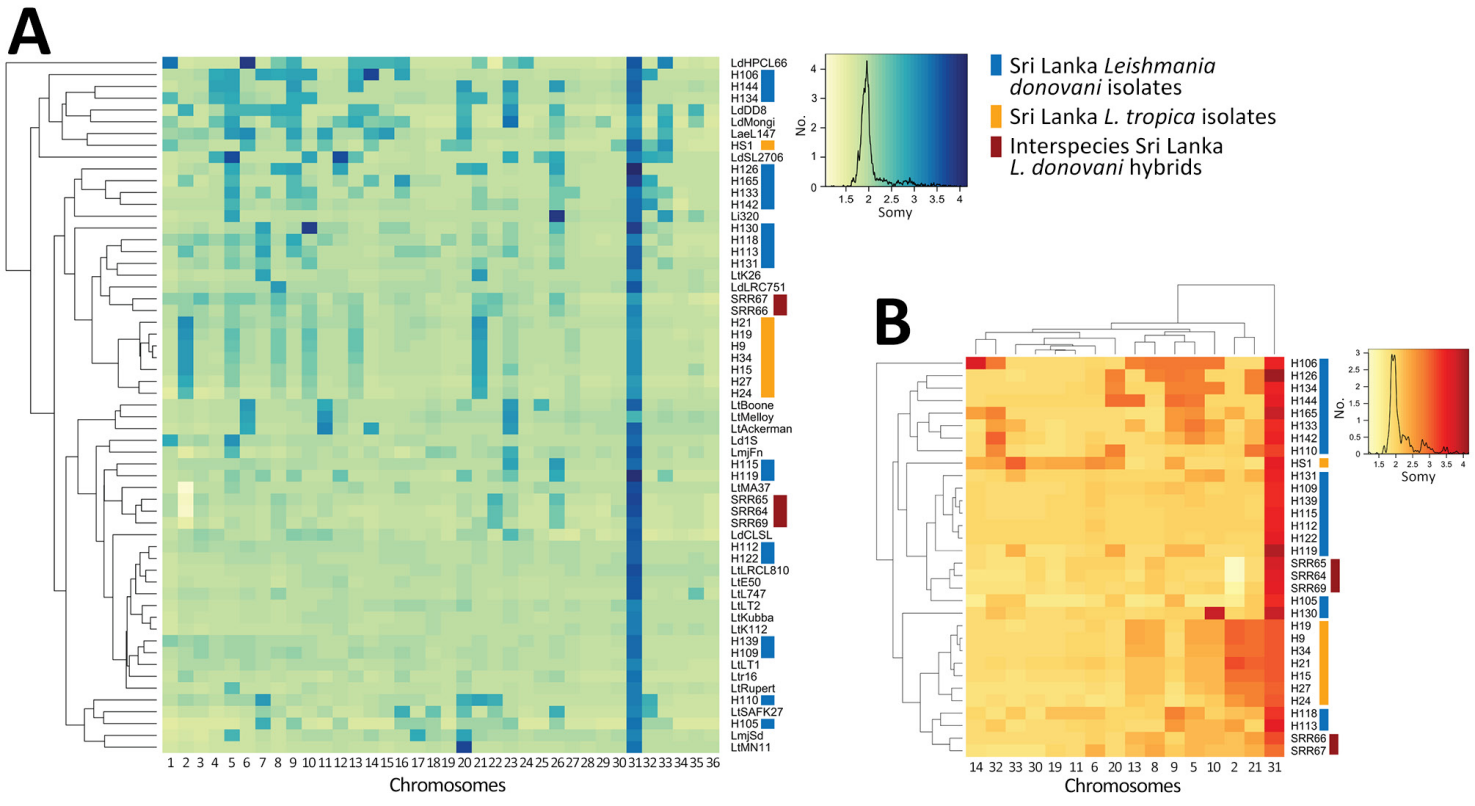
Highly conserved aneuploidy profiles within the cutaneous *Leishmania tropica* clade from Sri Lanka. A) Heat map visualization of *Leishmania* abnormal chromosome numbers in 27 patient isolates from Sri Lanka and 32 previously described strains or hybrids. *L. tropica* isolates from Sri Lanka are labeled with orange lines and *L. donovani* isolates from Sri Lanka are labeled with blue lines. B) Subset of the data shown in panel A highlighting only chromosomes that are polysomic in >1 isolate. Chromosome copy values were inferred from whole-genome analysis normalized read depth after mapping to the *L. tropica* L590 reference genome available on TriTrypDB (https://www.tritrypdb.org), with the assumption that all samples have a 2n DNA content. Somy, abnormal chromosome numbers.

NGS and MLST revealed that 19 genomes sequenced in this study belonged to the *L. donovani* complex and 8 to *L. tropica*. This finding was observed in every single locus investigated by MLST (Figure 2; Appendix 1 Figures 2, 3). Of note, the *ICD* gene sequence of the single MCL isolate, HS1, is more similar to *L. tropica* strains from the Middle East (including LtKubba from Syria, and LtLT1 and LtLT2 from Lebanon) than to isolates from Sri Lanka (Figure 2) (*8*,*18*).

### Chromosome Copy Profiles

Genome-wide read depth analysis revealed highly conserved chromosome copy profiles among the 7 Sri Lanka *L. tropica* CL isolates (H9, H15, H19, H21, H24, H27, and H34), and a divergent pattern in the single MCL isolate, HS1 (Figure 3, panels A, B). Polysomy of chromosome 31 (>3 copies) was seen in all samples. This polysomy was commonly observed in previous studies on *Leishmania* spp (*19*,*20*).

Trisomy (2.4–3.5 copies) of chromosomes 2, 21, and 31 and near-trisomy (2.2–2.4 copies) of chromosomes 5, 8, 10, and 13 were detected in *L. tropica* isolates from Sri Lanka except for HS1, which displayed trisomic chromosomes 6, 9, 11, 14, 19, 20, 30, 32, and 33 (Figure 3, panel B). In comparison, *L. donovani* clinical isolates from Sri Lanka displayed a more heterogenous distribution of chromosome copy values with varied karyotypes (Figure 3, panels A, B). Of the 3 previously reported *L. donovani–L. tropica* hybrids, SRR67 and SRR66 showed multiple near-trisomy matches with the *L. tropica* isolates from Sri Lanka on chromosomes 5, 8, 10, and 13. Of note, near-monosomy of chromosome 2 (1.2–1.4 copies) was detected in SRR64, SRR65, and SRR69 (Figure 3, panel B).

### Genome-Wide Zygosity Profiles and Phylogenetic Network Analysis

**Figure 4:**
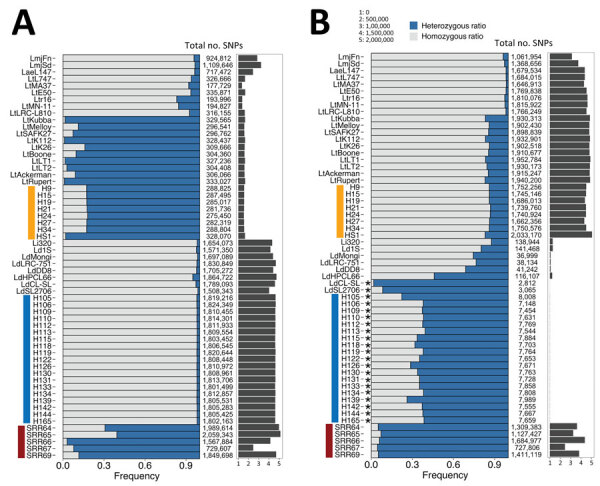
Frequency of genomewide heterozygous and homozygous SNPs in all genomes analyzed and presented as percent stacked bars after mapping sequencing reads from *Leishmania* spp. isolates from Sri Lanka. A) Mapped to *L. tropica* L590. B) Mapped to *L. donovani* cutaneous leishmaniasis reference genome. The total number of SNPs detected using the PAINT software suite (*16*) is shown to the right of the main plot. Vertical bars at left: blue, *L. donovani*; orange, *L. tropica*; red, interspecies hybrids. Asterisks, the biased heterozygosity profiles of *L. donovani* genomes that are highly similar to the reference genome (LdCL-SL), resulting in a low number of SNPs. SNP, single-nucleotide polymorphism.

The total number of SNPs per sample ranged from 117,729 (in LtMA37 WGS) to 2,059,343 (in SRR65 WGS) within the sample cohort after mapping reads to the *L.* *tropica* L590 reference genome (Figure 4 panel A). The 3 previously reported *L. donovani–L. tropica* hybrids showed low homozygosity (<0.12; SRR66–69). In contrast, most of the SNPs in the *L. donovani* isolates from Sri Lanka (H105–165) and in the intraspecies *L. donovani* hybrid LdHPCL66, identified in the Himachal Pradesh province of India, were homozygous (>0.98) (Figure 4, panel A) (*10*). Highly skewed heterozygosity was detected in most of the *L. tropica* genomes because they are genetically more similar to the *L. tropica* L590 reference genome than to *L. donovani*. For comparison, we also mapped reads to the *L. donovani* CL–Sri Lanka reference genome (Figure 4, panel B). As we expected for *L. donovani*–*L. tropica* progeny, both the genome-wide SNP frequencies and absolute numbers in the previously reported hybrids SRR66–69 were largely unaffected by the reference genome used for mapping (Figure 4). All *L. donovani* laboratory strains showed high ratios of homozygous SNPs.

**Figure 5:**
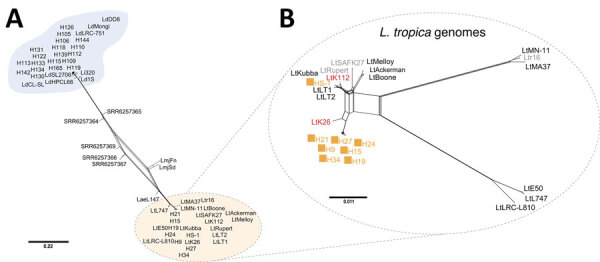
Phylogenetic network of all *Leishmania* isolates from Sri Lanka and Old World strains analyzed and visualized as a splits tree built using genomewide single-nucleotide polymorphisms in SplitsTree 6 (*17*). A) The cluster of the *L. donovani* complex is found at the top of the tree (blue) and the *L. tropica* are found at the bottom (orange). B) Phylogenetic network analysis of only the *L. tropica* genomes in our dataset. Orange squares, *L. tropica* isolates from Sri Lanka (HS1, H9–34); black font, Middle Eastern *L. tropica*; gray font, Azerbaijan (SAFK27) (*8*), Morocco (Ltr16) (*21*), and Afghanistan (Rupert) (*8*); red font, Indian *L. tropica* (K26 and K112) (*8*). Scale bar represents nucleotide substitutions per position.

We generated a network splits tree by using whole-genome SNPs for all the 59 *Leishmania* genomes, highlighting the separation between the *L. donovani* and *L.tropica* isolates from Sri Lanka, with the hybrids found in the middle point of the tree (Figure 5, panel A). A more detailed analysis by using only the data from the *L. tropica* genomes revealed 2 different subclusters of *L. tropica* in Sri Lanka. Although HS1 co-clusters with strains found in Syria (LtKubba) (*8*) and Lebanon (LtLT1 and LtLT2) (*18*) (Appendix 1 Figure 4, panels A, B), the other *L. tropica* from Sri Lanka form a discrete subgroup more genetically similar to the LtK26 strain from India (Figure 5, panel B). This finding corroborated the phylogenetic profile observed by the MLST analysis by using the *ICD* gene sequence (Appendix 1 Figure 3, panel C). Identification of *L. tropica* phylogenetic groups confirms worldwide *L. tropica* populations described by a previous comparative genomics study (*18*). We further expand on those findings by suggesting an additional population, consisting of LtK26 strain from India (*8*) and the H9–34 *L. tropica* from Sri Lanka.

### Genomewide Genetic Variations and Shared Ancestries

**Figure 6:**
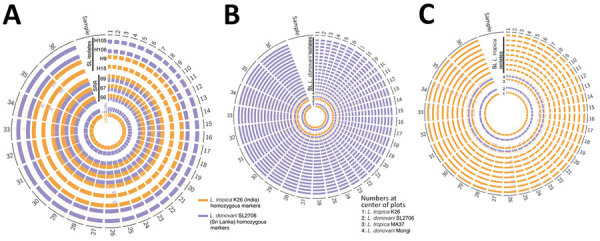
*Leishmania tropica* and *L. donovani* strains from the Indian subcontinent that share genetic markers with interspecies hybrids found in Sri Lanka. A) Circos plot (*22*) representation of the inheritance pattern of all genome-wide homozygous single-nucleotide polymorphism differences between strains *L. tropica* K26, found in India, and *L. donovani* SL2706, found in Sri Lanka. Each concentric circular track depicts parental allele contribution in the different genomes. Chromosomes are separated by white radial lines with chromosome numbers shown on the outermost circle. Representative genomes of the 2 species from Sri Lanka are shown, *L. tropica* (H9 and H15) and *L. donovani* (H105 and H106). *L. donovani* interspecies hybrids (SRR66–69) are examples of recent hybridization (*13*,*23*). Window size of 10 kb was used in the whole-genome sequencing analysis with the PAINT software suite (*17*) and reference genome LtrL590 available on TriTrypDB (https://www.tritrypdb.org). B) Circos plot (*22*) representing the allelic inheritance of LtK26 and LdSL2706 parental markers for the different *L. donovani* clinical isolates analyzed from Sri Lanka. C) Circos plot (*22*) representing the allelic inheritance of LtK26 and LdSL2706 parental markers for the different *L. tropica* clinical isolates analyzed from Sri Lanka. *L. donovani* Mongi (India) and *L. tropica* MA37 (Jordan) genomes are shown as references of nonmixed species genomes. SL, Sri Lanka.

**Figure 7:**
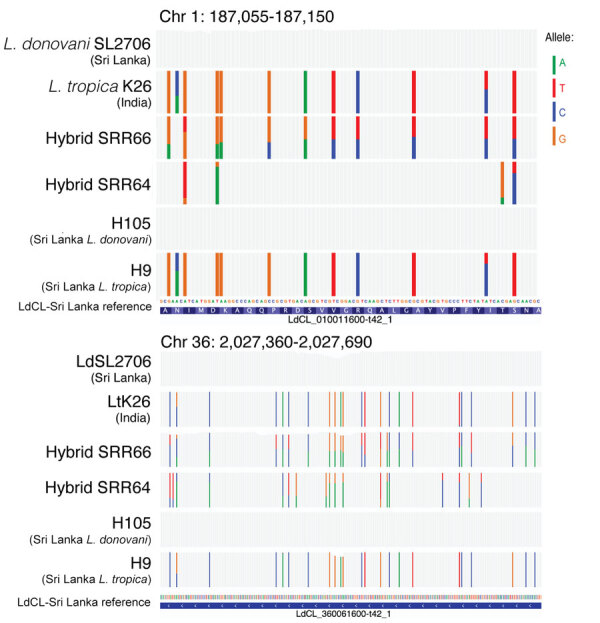
Representative nucleotide-level visualization of the inheritance of parental allelic markers on chromosomes 1 (upper panel) and 36 (lower panel) in *L. donovani*–*L. tropica* hybrid SRR66, *L. donovani*–*L. major* hybrid SRR64, and isolates from Sri Lanka (H105 and H9). Coverage plots highlighting single nucleotide polymorphisms were generated by using the integrative genomics viewer (https://igv.org), a match with the reference genome is represented as gray bars (*24*).

From the list of SNPs identified by using the PAINT software suite (*16*), we selected the 1,354,425 homozygous alleles that were different between *L. tropica* K26 and *L. donovani* SL2706 as representatives of the 2 clades found in Sri Lanka. We tracked their inheritance in hybrids SRR66, SRR67, and SRR69 and in the 27 Sri Lanka isolates. *L. donovani–L. tropica* markers were remarkably heterozygous across the whole genome of the SRR66–69 hybrids, suggesting they have undergone recent hybridization with biparental contribution from *L. donovani* and *L. tropica* in all 36 chromosomes (Figure 6, panel A). The number of SNPs shared with the *L. tropica* lineage from Sri Lanka was lower in the *L. donovani*–*L. major* hybrids (SRR64–65) (Appendix 1 Figure 5); only 31.5% of the heterozygous SNPs were explained by the tested *L. donovani–L. tropica* admixture compared with 70% in the *L. donovani–L. tropica* hybrids (SRR66–69). Visual inspection at the single-nucleotide level confirmed the extensive heterozygosity of *L. donovani–L. tropica* markers in the SRR66–69 hybrids for most of the sites analyzed, which was not as evident in the SRR64–65 hybrids (Figure 7; Appendix 1 Figure 6). The *L. donovani* (H105-H165) isolates from Sri Lanka showed low heterozygosity of selected parental markers and high similarity, and the *L. tropica* (HS1, H9-H34) isolates from Sri Lanka showed low heterozygosity of selected parental markers and high similarity to *L. tropica* from India lineages, evidence that these isolates have not experienced recent admixture of species (Figure 6, panels B, C).

### A2 Gene Variations

**Figure 8:**
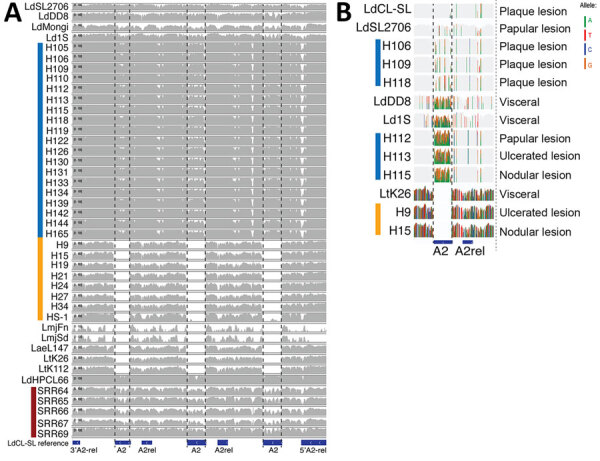
Coverage plots of whole-genome sequencing reads mapped to the reference strain *L. donovani* cutaneous leishmaniasis from Sri Lanka. A) The absence of reads on the A2 gene locus in the classic cutaneous species (*L. major* and *L. tropica*) indicates a truncated pseudogene sequence. B) Variable levels of sequence heterogeneity for the A2 gene are observed among the different *L. donovani* clinical isolates from Sri Lanka. Plaque skin lesion phenotype was associated with A2 gene sequences that are highly similar to the reference. SL, Sri Lanka.

*L. donovani* and the hybrids from Sri Lanka contain the full A2 gene sequences of all 4 annotated copies in the *L. donovani* CL–Sri Lanka reference genome on chromosome 22. Read depths in *L. tropica* isolates from Sri Lanka were virtually zero in those positions (Figure 8, panel A), except for HS1, which contained very low partial coverage of the A2 gene, indicating a truncated gene sequence. Of note, isolates H106, H109, and H119 causing plaque lesions in patients were the only isolates from Sri Lanka to carry A2 sequences that are highly similar to the *L. donovani* CL–Sri Lanka reference, which also induced a plaque lesion phenotype (Figure 8, panel B).

## Discussion

This study identified an endemic *L. tropica* causing leishmaniasis in Sri Lanka. The 7 patients with CL caused by *L. tropica* had never traveled overseas, and 1 had not traveled outside their district of residence for 5 years. The CL patients had been living in their area of residence either since birth or for 20–60 years. Thus, it is likely that these are autochthonous cases of CL caused by *L. tropica*.

This study identified MCL caused by the *L. tropica* isolate HS1 in Sri Lanka. The patient’s upper lip, nose, and the perinasal regions were affected similar to previously reported cases of MCL caused by *L. tropica* (*25*,*26*). This MCL patient had traveled to Dubai and Iraq 8 years before the diagnosis and had no history of cutaneous leishmaniasis. The time elapsed since the first appearance of symptoms to diagnosis was ≈8 months (Appendix 2 Table 4). Those facts suggest that either the patient began experiencing symptoms 7 years after a possible exposure during overseas travel or they acquired the infection in Sri Lanka.

The 7 cutaneous *L. tropica* isolates from Sri Lanka (H9–H34) were collected in southern Sri Lanka and belong to a genetically conserved subgroup that only co-clustered with *L. tropica* LtK26 from India. In contrast, the MCL-associated HS1 strain was isolated from a different geographic area and displayed a divergent chromosome copy profile more similar to *L. tropica* strains from Syria (LtKubba) and Lebanon (LtLT1 and LtLT2) than to the other *L. tropica* isolates from Sri Lanka (Figure 3; Figure 5, panel B), and a divergent genomewide SNP profile. Thus, it is possible that the infection was acquired in the Gulf region, and the mucosal manifestations might have become apparent years after the primary exposure.

Chromosome aneuploidy was evident among the study samples and the pattern differed between the *L. tropica* and *L. donovani* isolates. Noninteger chromosome copy values observed in this study are commonly found in *Leishmania* because of mosaic aneuploidy occurring in cultured parasite populations (*19*). Recent studies have shown that clonal growth in both sand flies and in vitro culture can by itself lead to major changes in chromosome copy values and karyotype diversity even after just 1 passage through the insect vector (*20*,*27*). In fact, concerning chromosome copy values, the *L. donovani*–*L. tropica* hybrids were among the most homogeneous genomes in our analysis.

From the data on patients’ residence and travel within Sri Lanka (Appendix 2 Table 4), it is evident that both *L. tropica* and *L. donovani* may not be restricted to a single administrative area at the micro level. Considering that *L. tropica* is predominantly causing anthroponotic leishmaniasis and that the patients with CL have traveled to other districts, further studies in additional locations and with other potential vectors would benefit the current understanding of leishmaniasis spread and genetic variability in Sri Lanka. In addition, zoonotic transmission of *L. tropica* through reservoirs such as rock hyraxes has been reported in other countries, and studies are needed to assess potential animal reservoirs of these parasites in Sri Lanka (*28*). Currently, there are no data available to dismiss either scenario, and both may represent valid and relevant routes of infection in the country. In addition to *L. tropica* causing cutaneous disease, its potential to visceralize in humans is known (*29*–*31*). Even though CL is the current predominant phenotype of leishmaniasis in Sri Lanka, the presence of *L. tropica* and *L. donovani*, both with potential visceralizing properties, raises concerns.

Studies on A2 gene expression in old world *Leishmania* spp. suggest its useful in determining species-specific organ tropism (*32*). Even though the pathogenicity of leishmaniasis is not fully understood, the understanding of CL lesions is they progress from an initial papule to a nodule or a plaque which can ultimately ulcerate. Several virulence factors were studied in the sequenced samples to understand the variability in *Leishmania* pathogenesis leading to different phenotypes. Because primarily cutaneous parasite species have a truncated A2 pseudogene, A2 is a gene family that occurs in *L. donovani* but not in *L. tropica* (*6*), a result seen in our study samples as well. A2 is necessary in the pathology of leishmaniasis and is known to aid in visceralization of the infection and survival within the host (*33*–*35*). Our analysis of the A2 gene in different phenotypes of *L. donovani* revealed marked differences in the plaque phenotype when compared with papules, nodules, and ulcers. A study conducted in Pakistan found that the clinical appearance of CL is not solely dependent on *L. tropica* genetic variations (*36*). This possibility could not be investigated in our study because most of the *L. tropica*–induced CL cases were ulcerated.

We have found there are >5 different populations of *Leishmania* in Sri Lanka: SL1, SL2A, SL2B, SL3 (*13*), and *L. tropica* from Sri Lanka (SL4). Our study has shown *L. tropica* has caused both CL and MCL in Sri Lanka. Those findings suggest further studies on differences in clinical phenotypes, potential vector species, and reservoirs of *Leishmania* species in Sri Lanka would be beneficial. Revisiting diagnostic and research approaches might be necessary in consideration of this species variability. Analysis by PCR and restriction fragment length polymorphism represent the inexpensive and easy laboratory methods available for distinguishing between the 2 species (*37*).

In conclusion, although this large *Leishmania* WGS dataset provides valuable insights, further whole-genome studies might lead to better understanding of the *Leishmania* species infecting people in Sri Lanka and might shed light on the extent of genetic heterogeneity of infective clades circulating in the country. This discovery is a turning point in understanding the pathology of leishmaniasis in Sri Lanka and may contribute to the development of more efficient diagnostics and treatments for CL and MCL caused by *L. tropica*.

Appendix 1 Additional information about autochthonous leishmaniasis caused by *Leishmania tropica*, identified by using whole-genome sequencing, Sri Lanka.

Appendix 2 Sequence read archive accession numbers for all the genomic data used in autochthonous leishmaniasis caused by *Leishmania tropica*, identified by using whole-genome sequencing, Sri Lanka.
