# Supplementary material for: Autochthonous Leishmaniasis Caused by Leishmania tropica, Identified by Using Whole-Genome Sequencing, Sri Lanka
Source: Emerg Infect Dis. 2024 Sep;30(9):1872–83. doi: 10.3201/eid3009.231238 (PMC11347009; doi:10.3201/eid3009.231238)
Supplement: Appendix 1 — Additional information about autochthonous leishmaniasis caused by Leishmania tropica, identified by using whole-genome sequencing, Sri Lanka. [file 23-1238-Techapp-s1.pdf]

Article DOI: https://doi.org/10.3201/eid3009.231238

EID cannot ensure accessibility for supplementary materials supplied by authors. Readers who have difficulty accessing supplementary content should contact the authors for assistance.

Autochthonous Leishmaniasis Caused by *Leishmania tropica*, Identified by Using Whole-Genome Sequencing, Sri Lanka

Appendix 1

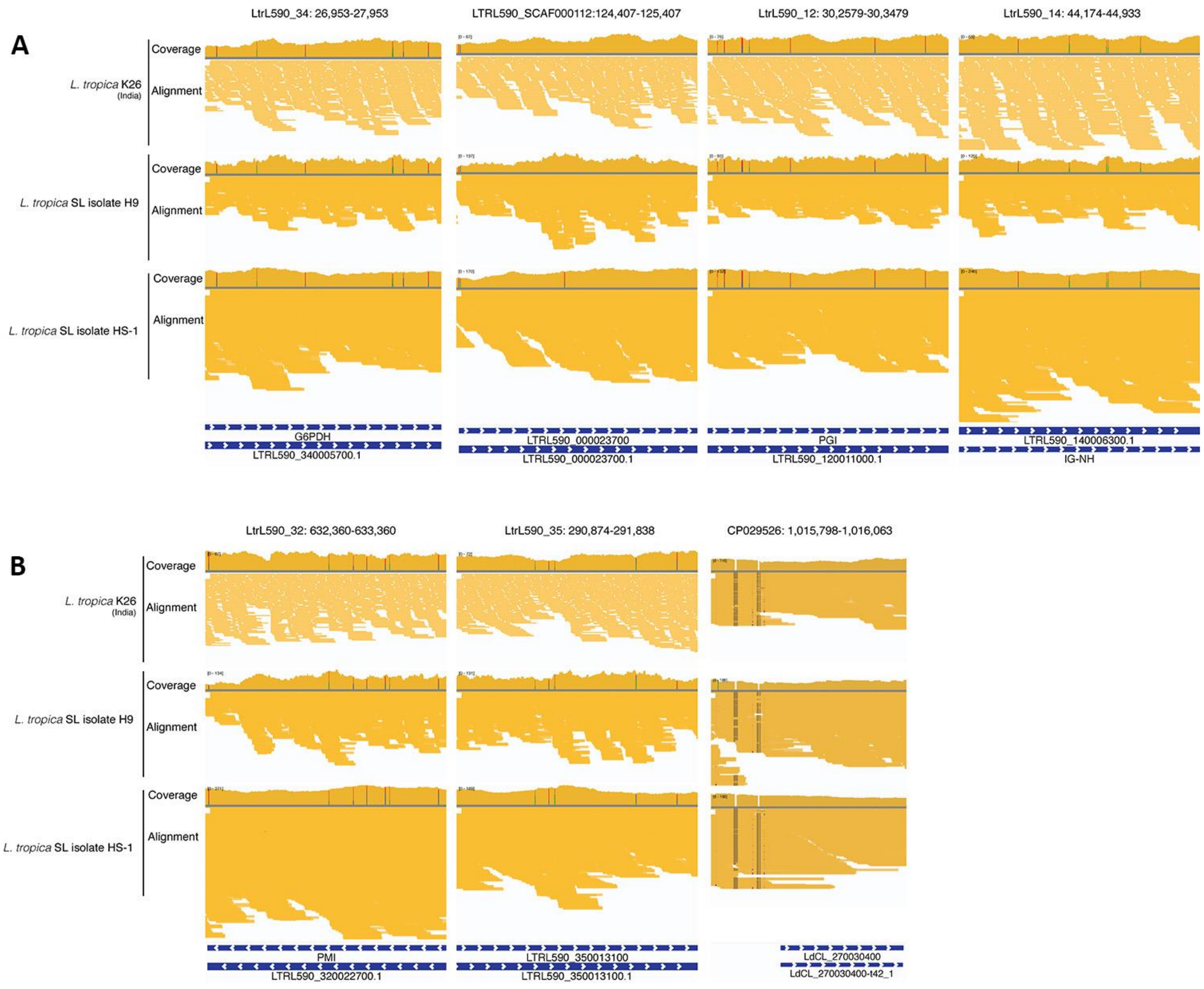

Appendix 1 Figure 1. NGS sequence read alignment to loci used for MLST in this study. Each panel depicts the different reference genome loci coordinates at the top, the coverage plot (upper graph) and pileup visualization of read mapping (lower graph) to the *L. tropica* L590 reference (for genes G6PD, ICD, GPI, IGNH, PMI and AST) or the LdSL-CL reference (for ITS) genomes.

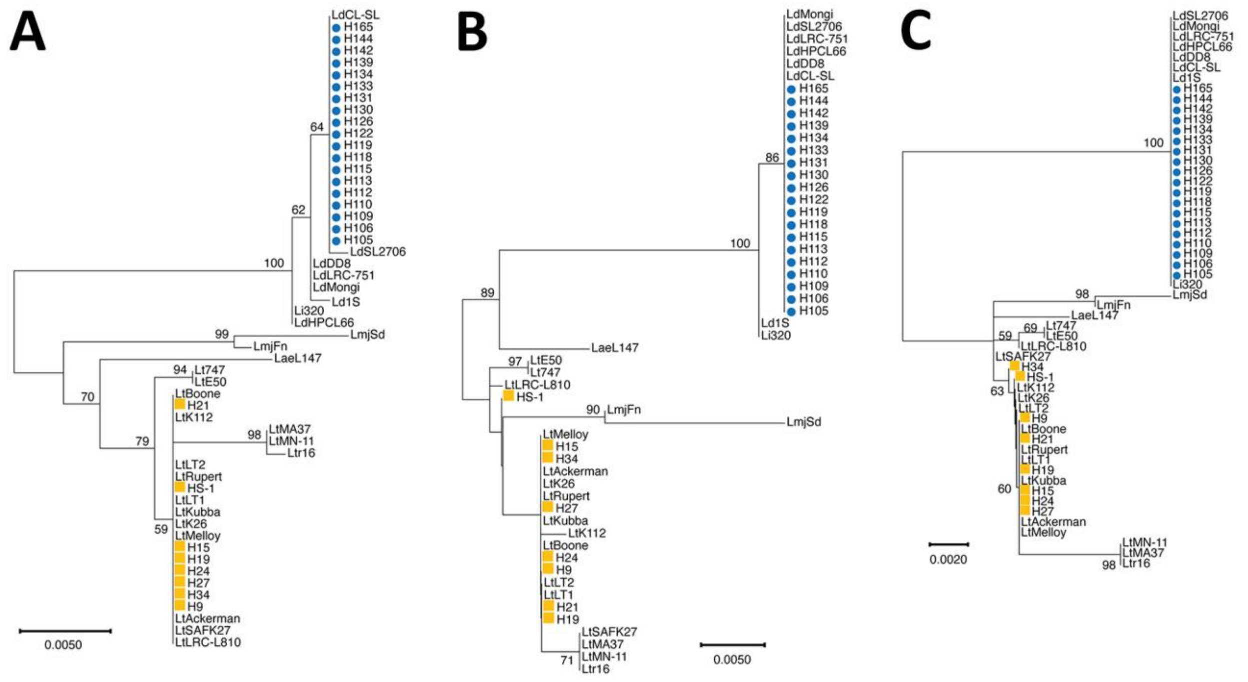

Appendix 1 Figure 2. Phylogenetic analysis of Sri Lanka *Leishmania* isolates and Old World *Leishmania* spp. by MLST using nucleotide gene sequences of aspartate aminotransferase (*AST*), phosphomannose isomerase (*PMI*) and inosine-guanine nucleoside hydrolase (*IGNH*). Sri Lanka isolates (H and HS) co-cluster with either *L. donovani* (blue circles) or *L. tropica* (orange squares). Maximum-likelihood method and Tamura-Nei model were performed for phylogenetic analysis using MEGA X software (*1*). The trees with the highest log likelihood are presented from 1000 bootstrap replicates. Bootstrap percentages higher than 55% are shown for each branch and scale bar represents a phylogenetic distance of mutations per position.

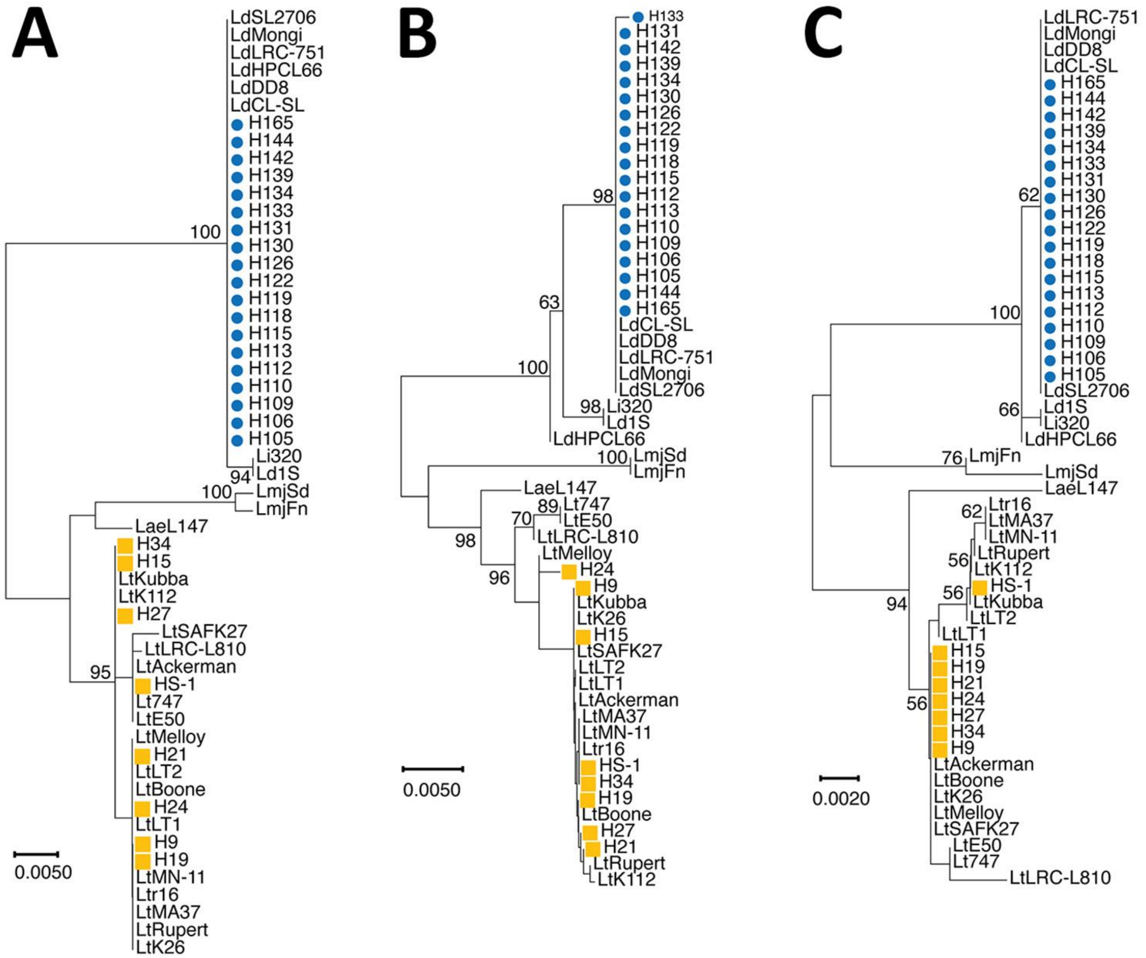

Phylogenetic analysis of Sri Lanka patient isolates and different *Leishmania species* and strains using the sequences of the A) glucose-6-phosphate 1-dehydrogenase (*G6PD*), B) glucose-6-phosphate isomerase (*GPI*) and C) isocitrate dehydrogenase precursor (*ICD*). Sri Lanka isolates (H and HS) form two groups, one co-cluster with *L. donovani* (blue circles) and the other with *L. tropica* (orange squares). Maximum-likelihood method and Tamura-Nei model were performed for phylogenetic analysis using MEGA X (*14*). For each gene, the phylogenetic tree with the highest log likelihood is presented from 1000 bootstrap replicates. Bootstrap percentages higher than 55% are shown for each branch and scale bars are mutations per site.

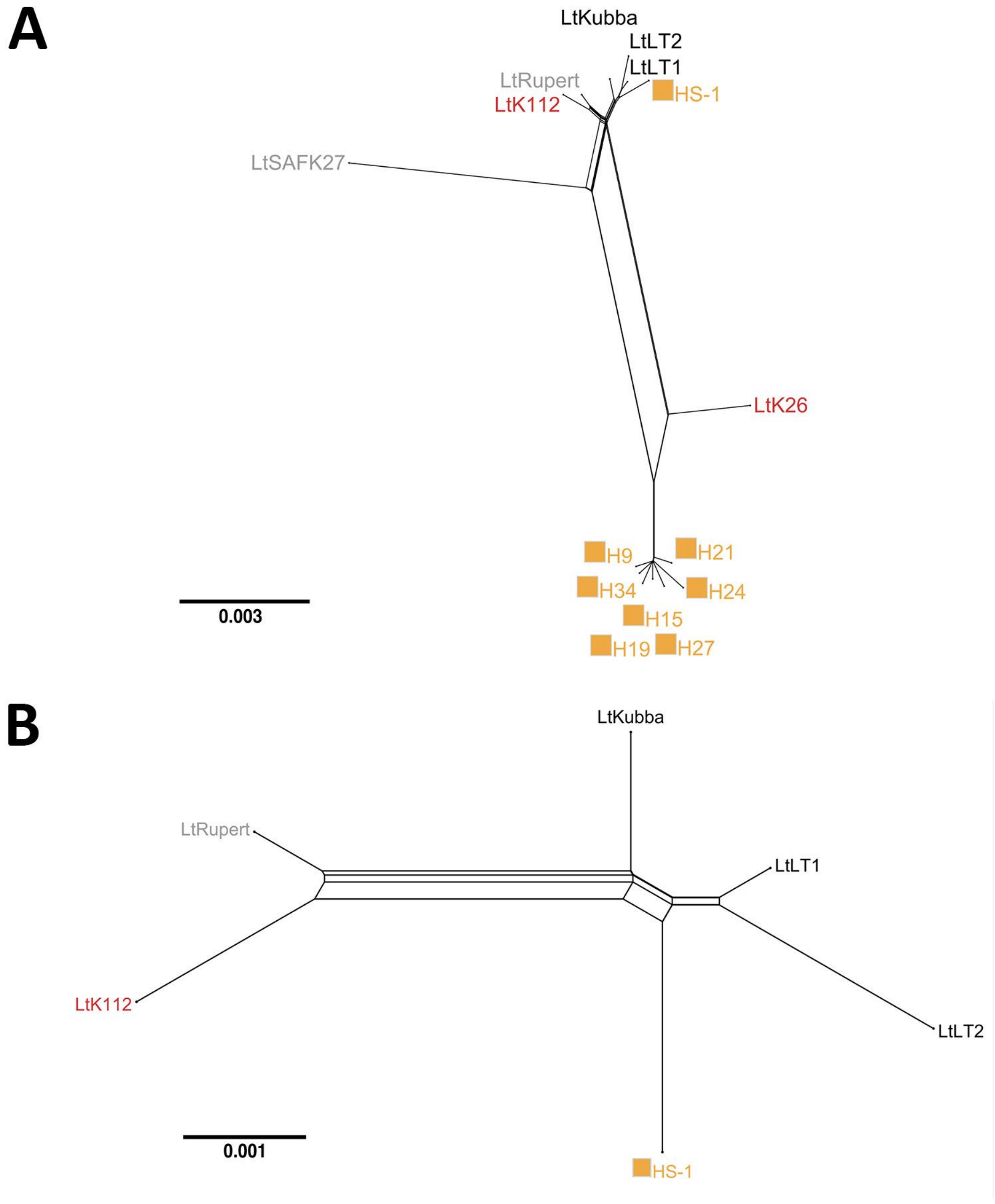

Appendix 1 Figure 4. Phylogenetic network of subgroups of *L. tropica* found in the Indian subcontinent and in the Middle East visualized as a splits tree built using genome-wide SNPs in SplitsTree 6 (*2*). Networks are shown for *L. tropica* genomes co-clustering with isolates H9–34 or HS-1 (A) or in more detail only for those co-clustering with HS-1 (B). Similar analysis including all the *L. tropica* genomes investigated in this work is shown in Figure 5B. Orange squares: Sri Lanka *L. tropica* isolates (HS1, H9–34); Red font: Indian *L. tropica* (K26 and K112) (*3*); Black font: Middle Eastern *L. tropica*; Grey font: Azerbaijan (SAFK27) (*3*) and Afghanistan (Rupert) (*3*).

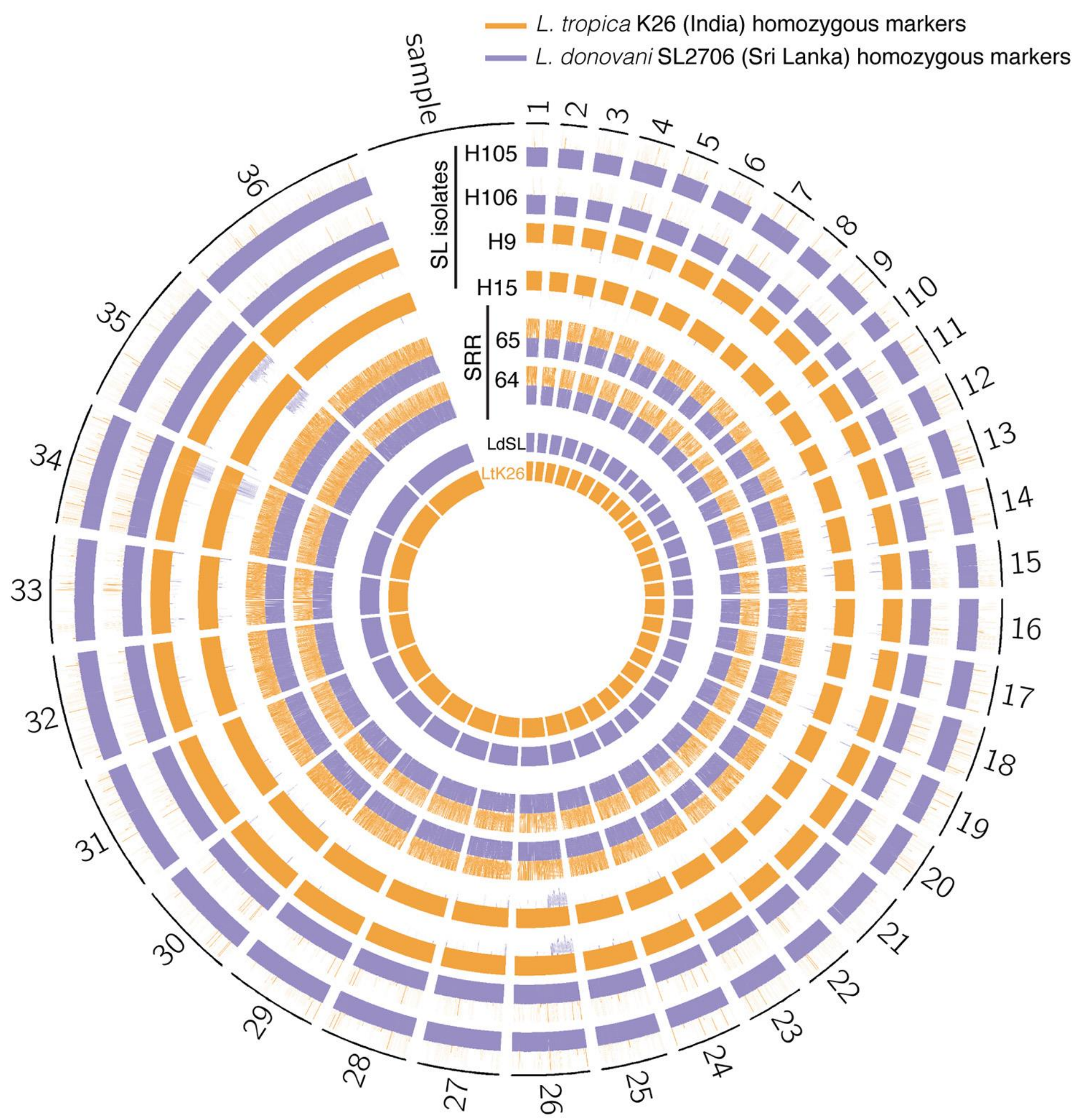

Appendix 1 Figure 5. Contribution of allelic homozygous differences between Indian *L. tropica* (Lt) K26 and Sri Lankan *L. donovani* (Ld) SL2706 in Sri Lanka clinical isolates and *L. donovani-L. major* hybrids (SRR64–65)**.** The two hybrid genomes shown were previously described as *L. donovani-L. major* interspecies progeny. SNP analysis was performed using PAINT software (*4*). Circos plots were generated using Circos (*5*).

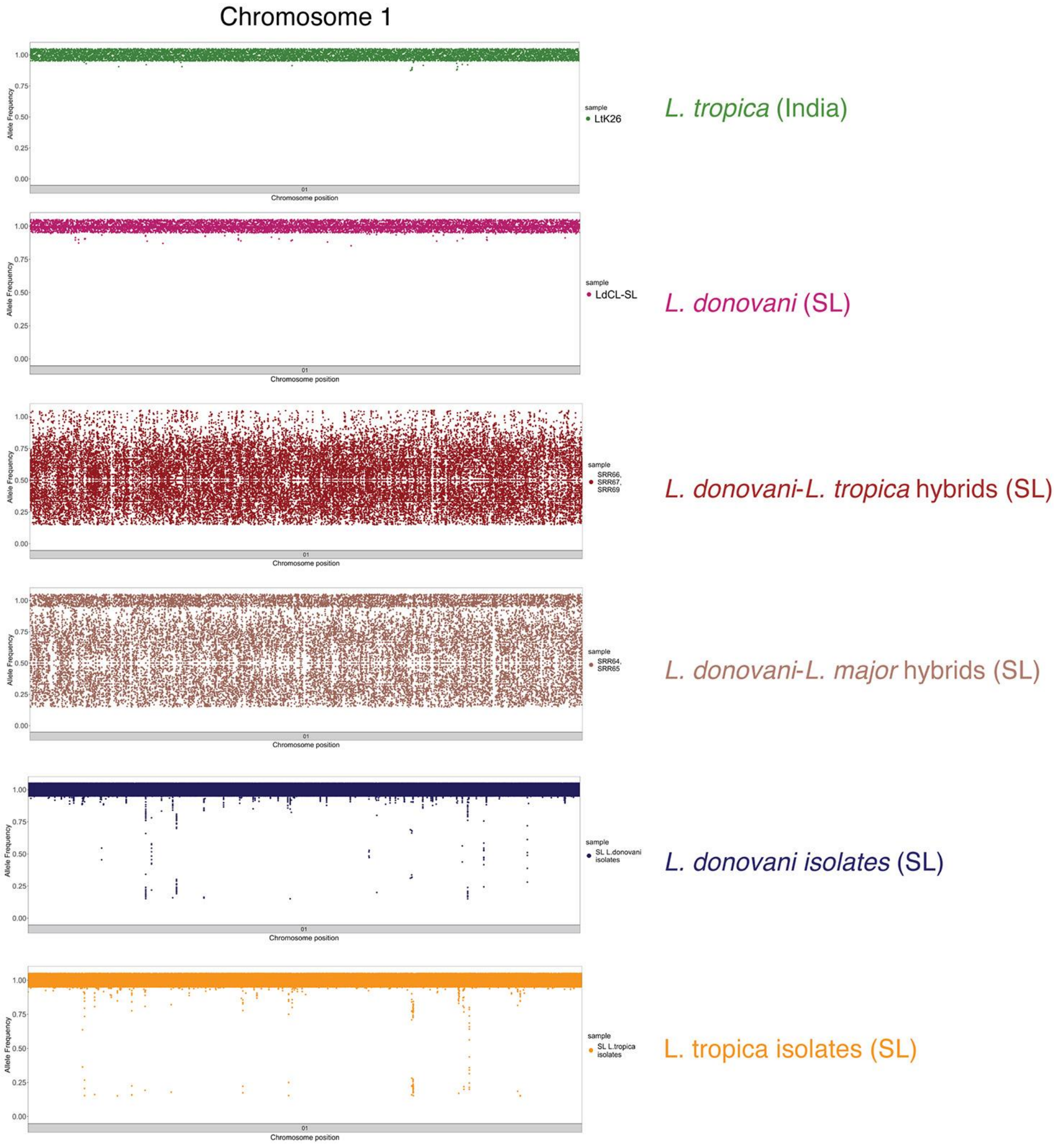

Appendix 1 Figure 6. Scatter plot visualization of the frequencies of alleles on chromosome 1 that are homozygous SNPs in both LtK26 (green) and in LdSL2706 (magenta) regardless of whether SNPs were identical or distinct between the two strains using the *L. tropica* L590 reference genome. Frequency of alleles on the same positions are shown for Sri Lanka *L. donovani* (dark blue) and Sri Lanka *L. tropica* (orange) isolates and previously reported *L. donovani*-*L. major* hybrids (light brown) and *L. donovani*-*L. tropica* hybrids (dark brown).

References

1. Kumar S, Stecher G, Li M, Knyaz C, Tamura K. MEGA X: Molecular evolutionary genetics analysis across computing platforms. Mol Biol Evol. 2018;35:1547–9. PubMed https://doi.org/10.1093/molbev/msy096

2. Huson DH, Bryant D. Application of phylogenetic networks in evolutionary studies. Mol Biol Evol. 2006;23:254–67. PubMed https://doi.org/10.1093/molbev/msj030

3. Iantorno SA, Durrant C, Khan A, Sanders MJ, Beverley SM, Warren WC, et al. Gene expression in *Leishmania* is regulated predominantly by gene dosage. MBio. 2017;8:1–20. PubMed https://doi.org/10.1128/mBio.01393-17

4. Shaik JS, Dobson DE, Sacks DL, Beverley SM. *Leishmania* sexual reproductive strategies as resolved through computational methods designed for aneuploid genomes. Genes (Basel). 2021;12:1–16. PubMed https://doi.org/10.3390/genes12020167

5. Krzywinski M, Schein J, Birol I, Connors J, Gascoyne R, Horsman D, et al. Circos: an information aesthetic for comparative genomics. Genome Res. 2009;19:1639–45. PubMed https://doi.org/10.1101/gr.092759.109
